# Insulin Deficiency Increases Sirt2 Level in Streptozotocin-Treated Alzheimer’s Disease-Like Mouse Model: Increased Sirt2 Induces Tau Phosphorylation Through ERK Activation

**DOI:** 10.1007/s12035-022-02918-z

**Published:** 2022-06-15

**Authors:** Chunyu Zhou, Cha-Gyun Jung, Mi-Jeong Kim, Atsushi Watanabe, Mona Abdelhamid, Ferdous Taslima, Makoto Michikawa

**Affiliations:** 1 Department of Biochemistry, Nagoya City University Graduate School of Medical Sciences, Nagoya, Aichi 467-8601 Japan; 2 Department of Food & Biotechnology, Korea University, Sejong, 30019 South Korea; 3 Laboratory of Research Advancement, Research Institute, National Center for Geriatrics and Gerontology, Obu, Aichi 474-8511 Japan

**Keywords:** Alzheimer’s disease, Streptozotocin, Insulin deficiency, Sirtuin 2, Tau phosphorylation, ERK

## Abstract

Accumulating evidence suggests that insulin deficiency is a risk factor for Alzheimer’s disease (AD); however, the underlying molecular mechanisms are not completely understood. Here, we investigated the effects of insulin deficiency on AD-like pathologies using an insulin-deficient amyloid-β (Aβ) precursor protein (APP) transgenic mouse model (Tg2576 mice). Female Tg2576 mice were injected intraperitoneally with streptozotocin (STZ) to induce insulin deficiency, and their body weights, serum glucose levels, and serum insulin levels were evaluated. STZ-treated mice showed exacerbated Aβ accumulation, tau hyperphosphorylation, glial activation, neuroinflammation, and increased Sirt2 protein levels in the brain, as determined by two-dimensional gel electrophoresis (2-DE) coupled with liquid chromatography–tandem mass spectrometry (LC–MS/MS) and Western blotting. Furthermore, our in vitro experiments revealed that insulin depletion or interleukin-6 treatment increased Sirt2 protein levels in both Neuro2a and Neuro2a-P301L cells. The overexpression of Sirt2 in these cells induced tau hyperphosphorylation through extracellular signal-regulated kinase (ERK) activation. Conversely, Sirt2 knockdown reversed tau hyperphosphorylation in these cells. We showed for the first time that Sirt2 is upregulated in the brains of STZ-treated Tg2576 mice and is involved in tau phosphorylation through ERK activation. Our findings suggest that Sirt2 is a promising therapeutic target for the treatment of AD.

**Supplementary Information:**

The online version contains supplementary material available at 10.1007/s12035-022-02918-z.

## Introduction

Alzheimer’s disease (AD) is a major neurodegenerative disease characterized by the presence of extracellular senile plaques, intraneuronal neurofibrillary tangles, neuroinflammation, and neuronal loss [1]. Amyloid-β (Aβ) is generated from the Aβ precursor protein (APP) by β- and γ-secretases, resulting in the subsequent formation of Aβ plaques [2]. Intraneuronal neurofibrillary tangles are composed of aggregated hyperphosphorylated tau proteins [3].

Impairment in insulin signaling, including insulin deficiency (type 1 diabetes mellitus, T1DM) and insulin resistance (type 2 diabetes mellitus, T2DM), affect not only peripheral tissues but also brain functions that are involved in AD pathologies [4, 5]. Recently, preclinical and clinical studies have suggested that diabetes is a risk factor for AD: the levels of insulin and its receptors were found to be reduced in the brains of patients with AD, suggesting impairments in insulin signal transduction [6, 7], which are associated with memory and cognitive deficits and late-onset AD [8]. Therefore, the disruption of insulin signaling is thought to be a common feature of both AD and diabetes. Indeed, intranasal administration of insulin has been shown to partially improve cognition in AD [9, 10] and reduce AD-like pathology in 3xTg-AD mice [11] suggesting that it may be used for the treatment of AD [12].

T1DM and T2DM can be modeled in animals by administration of streptozotocin (STZ), which induces pancreatic β-cell destruction and is widely used in studies of insulin function [13–16]. Insulin signaling has been reported to regulate Aβ levels and tau phosphorylation in the brains of APP transgenic mouse models. Depletion of insulin in these mice by administration of STZ has been shown to increase Aβ accumulation in the brain, which leads to cognitive impairment [17, 18]. This Aβ accumulation was caused by both an increase in Aβ generation and an inhibition of its degradation through downregulation of the Aβ-degrading enzyme insulin-degrading enzyme (IDE) [17, 18]. In addition to Aβ pathology, several lines of evidence have shown that insulin deficiency in the brains of STZ-treated APP transgenic mice increases tau phosphorylation. Tau phosphorylation is regulated by tau kinases, such as glycogen synthase kinase-3β (GSK3β), extracellular signal-regulated kinase (ERK), c-Jun N-terminal kinase (JNK), and p38 which are considered to be major physiological and pathological tau kinases [19], and by protein phosphatase (PP)-1 and PP2A, which have strong tau dephosphorylation activities [20]. While several studies have shown that insulin signaling regulates Aβ and tau pathologies [4, 18, 21], the detailed molecular mechanism by which insulin deficiency affects AD pathologies is poorly understood.

Sirtuins (Sirt1–7) are class III histone deacetylases that regulate critical biological processes, including metabolism, cell growth, cellular stress responses, and aging [22]. Sirt1 is thought to be neuroprotective, and its expression is reduced in neurodegenerative disorders; it is involved in AD-like physiopathogenic mechanisms, including neuroinflammation, APP processing, and mitochondrial dysfunction [22, 23]. In contrast to Sirt1, Sirt2 may contribute to neurodegeneration given its high expression levels in neurodegenerative disorders, including AD and Parkinson’s disease (PD), and the Sirt2 single-nucleotide polymorphism rs10410544 has been reported to increase the risk of AD [22]. Moreover, Sirt2 inhibition reduced α-synuclein toxicity in models of PD and conferred neuroprotection in models of Huntington’s disease [24, 25]. It has also been reported that Sirt2 inhibition improves cognitive function, reduces neuroinflammation, and decreases Aβ levels in vivo [26, 27]; thus, the inhibition of Sirt2 may be a promising therapeutic strategy in neurodegenerative disorders. Although this enzyme is known to be involved in neurodegeneration, its role in AD pathologies under conditions of insulin deficiency remains unknown.

In this study, we established a combined insulin deficiency/AD-like model in Tg2576 APP transgenic mice by intraperitoneal (i.p.) injection of STZ. We assessed how insulin deficiency affects this mouse model and performed two-dimensional gel electrophoresis (2-DE) coupled with liquid chromatography–tandem mass spectrometry (LC–MS/MS) analysis to identify the potential key proteins that may be involved in AD-like pathologies. Our findings corroborate the impact of insulin deficiency on AD-like pathologies and provide evidence that Sirt2 plays a major role in this context, suggesting that this enzyme may be a promising target in the development of AD treatment.

## Materials and Methods

### Experimental Animals

Female Tg2576 mice harboring human APP695 with the Swedish mutation KM660/671NL were purchased from Taconic (Germantown, NY, USA). We chose only female mice in this study because they can be group-hosed, whereas male Tg2576 mice would have required individual housing to avoid injuries from fighting, and individual housing could be stressful to them [28]. The mice were housed under a 12-h light/dark cycle with ad libitum access to food and water. Three-month-old Tg2576 mice were randomly divided into vehicle (*n* = 11) and STZ (*n* = 11) groups; the mice were fasted for 4 h and received i.p. injections of vehicle or STZ (50 mg/kg, dissolved in 0.1 M sodium citrate solution), respectively, once a day for 5 consecutive days. During the experiment, the body weight and blood glucose levels of the mice were monitored at 1, 4, 8, 12, and 16 weeks after STZ administration. Blood was extracted from the tail for the measurement of glucose levels using a glucometer. At 16 weeks after STZ administration, serum insulin levels were determined using an enzyme-linked immunosorbent assay (ELISA) kit (FUJIFILM Wako Pure Chemical Corporation, Osaka, Japan). All experiments were carried out in conformity with the National Institute of Health Guide for the Care and Use of Laboratory Animals and were approved by the Nagoya City University Institutional Care and Use of Laboratory Animals Committee.

### Immunohistochemistry

Sixteen weeks after STZ administration, the mice were anesthetized and perfused transcardially with cold phosphate-buffered saline (PBS), and their brains were quickly removed. One hemibrain (*n* = 8) was fixed in 4% paraformaldehyde and embedded in paraffin. Coronal brain sections were cut (10 μm thick) and stored at room temperature (RT) until immunohistochemical analysis. Briefly, paraffin sections were deparaffinized, rehydrated, boiled in 0.01 M trisodium citrate (pH 6.0) for 5 min, and treated with 3% hydrogen peroxide in PBS for 10 min. After washing with PBS, the sections were incubated with anti-Aβ antibody (82E1, 1:100, IBL, Gunma, Japan) overnight at 4 °C. Immunopositive signals were visualized using the ABC Elite kit (Vector Laboratories Inc., Burlingame, CA, USA), and images were obtained using the Axio Observer microscope (Carl Zeiss). Aβ plaques were quantified as the percentage of the immunostained area (positive pixels) divided by the examined area (total pixels) using ImageJ software (NIH, Bethesda, MD, USA). Immunofluorescence staining was assessed using the Opal 4-Color Manual IHC Kit (PerkinElmer, Waltham, MA, USA) according to the manufacturer’s instructions. Briefly, antigen-retrieved sections were treated with 0.3% hydrogen peroxide in MeOH to inactivate the intracellular peroxidases and then blocked with 10% goat serum in Tris-buffered saline (TBS) with 0.05% Tween 20 (TBS-T) for 20 min at RT. Sections were incubated with the according primary antibodies overnight at 4 °C: anti-Sirt2 (Sigma-Aldrich, St. Louis, MO, USA), anti-glial fibrillary acidic protein (GFAP) (Sigma-Aldrich), and anti-neuronal nuclei (NeuN) (Cell Signaling Technology, Danvers, MA, USA). The sections were then washed with TBS-T and incubated with Opal Polymer horseradish peroxidase (HRP) Ms + Rb for 30 min at RT. Signal amplification was performed by treatment with an Opal fluorophore working solution for 10 min at RT. Slides were boiled in AR buffer to strip primary-secondary-HRP complexes, allowing the use of subsequent primary antibodies. Nuclei were stained with DAPI (Thermo Fisher Scientific, Rockford, IL, USA). Images were obtained using the confocal fluorescence microscope SpinSR10 (Olympus, Tokyo, Japan). To quantify the Sirt2 fluorescence intensities, we randomly selected 2 fields (1 × 1 mm^2^ square per field) of the hippocampus and 4 fields (1 × 1 mm^2^ square per field) of the cortex, and the mean was used for statistical analysis. All images were analyzed using ImageJ software. Fluorescence intensities are expressed as arbitrary units (A.U.)

### Aβ ELISA

Brain Aβ levels were determined as described in a previous report [29]. Briefly, 6 remaining hemibrains were homogenized in TBS containing a protease inhibitor cocktail (Roche Diagnostics GmbH, Mannheim, Germany) and then the resulting homogenates were divided two groups. One group was used for Aβ ELISA (*n* = 6), and the other group was used for Western blot analysis (*n* = 6). For Aβ ELISA, the resulting homogenates were centrifuged at 100,000 rpm for 20 min at 4 °C. The supernatants were transferred and stored at − 80 °C until analysis and used for soluble Aβ determination. The resulting pellets were resuspended with 6 M guanidine hydrochloride, sonicated, and incubated for 1 h at RT. The homogenates were centrifuged at 100,000 rpm for 20 min at 4 °C, and the resulting supernatants were transferred and stored at – 80 °C until analysis and used for insoluble Aβ determination. To detect soluble and insoluble Aβ40 and Aβ42 levels, commercial Aβ ELISA kits (FUJIFILM Wako Pure Chemical Corporation) were used according to the manufacturer’s instructions. The obtained Aβ levels were normalized to brain tissue weight.

### RNA Extraction and Real-Time Polymerase Chain Reaction

Total RNA was extracted from the 2 remaining hemibrains and hemibrains from 3 mice (totally *n* = 5) using TRIzol (Invitrogen, Carlsbad, CA, USA) according to the manufacturer’s instructions. Total RNA (1 µg) was converted to complementary DNA (cDNA) using the iScript Select cDNA Synthesis kit (Bio-Rad, Hercules, CA, USA) in accordance with the manufacturer’s instructions. Quantitative real-time polymerase chain reaction (qRT-PCR) was performed using the GeneAce SYBR qPCR Mix (Nippon Gene, Tokyo, Japan) with the 7500 Fast Real-Time PCR System (Applied Biosystems, Grand Island, NY, USA). The mRNA expression levels of interleukin (IL)-6 and tumor necrosis factor (TNF)-α were analyzed using the comparative threshold cycle method and normalized with the corresponding expression of glyceraldehyde-3-phosphate dehydrogenase (GAPDH) as an endogenous control. Amplification was performed using the following primers: IL-6 forward, 5′-TGATGGATGCTACCAAACTGAT-3′, reverse, 5′-CTGTGACTCCAGCTTATCTCTT-3′; TNF-α forward, 5′-GGGCTTCCAGAACTCCAGG-3′, reverse, 5′-GCTCTCCACTTGGTGGTTT-3′; and GAPDH forward, 5′-GCATCTTCTTGTGCAGTGCC-3′, reverse, 5′-GAGAAGGCAGCCCTGGTAAC-3′.

### Brain Tissue Preparation for 2-DE

2-DE was performed as previously described [30]. Briefly, we pooled the cortical tissue of three vehicle- and three STZ-treated mice, and homogenized the pooled tissue of each group in a lysis solution containing 7 M urea, 2 M thiourea containing 4% (w/v) 3-[(3-cholamidopropyl)-dimethylammonium]-1-propanesulfonate (CHAPS), 1% (w/v) dithiothreitol (DTT), 2% (v/v) pharmalyte, and 1 mM benzamidine. Proteins were extracted by vortexing for 1 h at RT. After centrifugation at 15,000 g for 1 h at 15 °C, insoluble material was discarded and the soluble fraction was used for 2-DE. Protein loading was normalized using the Bradford assay. Immobilized pH gradient dry strips were equilibrated for 12 to 16 h with 7 M urea and 2 M thiourea containing 2% CHAPS, 1% DTT, and 1% pharmalyte, and loaded with 200 µg of sample. Isoelectric focusing (IEF) was performed at 20 °C using the Multiphor II electrophoresis unit and EPS 3500 XL power supply (Amersham Biosciences, Uppsala, Sweden) according to the manufacturer’s instructions. For IEF, the voltage was linearly increased from 150 to 3500 V over a period of 3 h for sample entry, followed by a constant 3500 V, with a complete focus after 96 kVh. Before the second-dimension run, strips were incubated for 10 min in equilibration buffer (50 mM Tris–Cl, pH 6.8, containing 6 M urea, 2% sodium dodecyl sulfate [SDS], and 30% glycerol), first with 1% DTT and second with 2.5% iodoacetamide. Equilibrated strips were inserted onto SDS–polyacrylamide gel electrophoresis (SDS-PAGE) gels (20 × 24 cm, 10–16%). SDS-PAGE was performed using the Hoefer DALT 2D system (Amersham Biosciences) following the manufacturer’s instructions. The 2-D gels were run at 20 °C for 1700 Vh.

### Image Analysis

The 2-D gels were stained with Coomassie G250. Quantitative analysis of digitized images was performed using the PDQuest (BioRad) software according to the protocols provided by the manufacturer. The quantification of each spot was normalized to the total valid spot intensity. Protein spots were selected for significant variation of expression (over two-fold) compared with the control sample.

### Protein Identification

Protein samples were analyzed using LC–MS/MS as previously described [31]. Briefly, proteins in the stained bands were reduced with 10 mM dithiothreitol at RT for 2 h and alkylated with 40 mM iodoacetamide in the dark at RT for 30 min. Each sample was digested with sequencing-grade modified trypsin (4 μg/mL; Trypsin Gold, Promega, Madison, WI, USA) in 40 mM NH_4_HCO_3_/10% acetonitrile overnight at 37 °C. The extracted peptides were then separated via nanoflow LC (Paradigm MS4, Michrom BioResources, Inc., Horsham, PA, USA) using a reverse phase C18 column (Magic C18, 0.2 × 50 mm; Michrom BioResources, Inc.). The LC eluent was coupled to a micro-ionspray source attached to the LCQ Advantage MAX mass spectrometer (Thermo Fisher Scientific). All MS/MS spectra were searched using the TurboSEQUEST algorithm within the BioWorks 3.2 software (Thermo Fisher Scientific).

### Cell Cultures

Primary cortical neurons were obtained from embryonic day 17 Wistar rats (Japan SLC, Inc. Shizuoka, Japan) and cultured as previously described [32]. Briefly, minced embryonic brain tissue was incubated in PBS containing 0.125% trypsin and 0.4 mg/mL DNase I for 15 min at 37 °C, followed by dissociation into single cells by pipetting. The dissociated cells were cultured in Neurobasal medium (Thermo Fisher Scientific) containing MACS NeuroBrew-21 without vitamin A (Miltenyi Biotec., Gladbach, Germany) and 0.1% bovine albumin fraction V solution (Thermo Fisher Scientific). Four days after plating, the cells were used for further experiments. Neuro2a cells, a mouse neuroblastoma cell line, and Neuro2a-P301L cells stably expressing the human 2N4R tau isoform and the repeat domain fragment with the P301L mutation were cultured in Dulbecco’s modified Eagle’s medium (DMEM; FUJIFILM Wako Pure Chemical Corporation) supplemented with 10% fetal bovine serum (FBS). Neuro2a-P301L cells were kindly provided by Dr. Akihiko Takashima (Gakushuin University, Japan) [33].

### Insulin and Il-6 Treatment

Neuro2a and Neuro2a-P301L cells were cultured in 10% FBS/DMEM medium for 24 h. After washing the cells with FBS-free DMEM, the cells were treated with or without 10 nM insulin in FBS-free DMEM medium for 24 h. In additional experiments, the cells were treated with or without 50 ng/mL IL-6 (FUJIFILM Wako Pure Chemical Corporation) in FBS-free DMEM medium for 30 min.

### Plasmid Transfection

A Myc-tagged Sirt2 plasmid (pCMV3-C-Myc) was purchased from Sino Biological Inc. (Beijing, China). Neuro2a and Neuro2a-P301L cells were transiently transfected with the Sirt2 or mock control vector using the Lipofectamine 3000 reagent (Thermo Fisher Scientific) in accordance with the manufacturer’s instructions. The effects were examined 48 h after transfection.

### RNA Interference

Sirt2 knockdown was performed using predesigned Stealth siRNA against Sirt2 (Sirt2 siRNA), and a Stealth siRNA negative control was used as control siRNA (Invitrogen). The Sirt2 siRNA sequences were as follows: sense, 5′-GCCAUCUUUGAGAUCAGCUACUUCA-3′; antisense, 5′-UGAAGUAGCUGA-UCUCAAAGAUGGC-3′. Primary cultured neurons, Neuro2a, and Neuro2a-P301L cells were transiently transfected with Sirt2 or control siRNA using Lipofectamine RNAiMAX (Invitrogen) in accordance with the manufacturer’s instructions. Seventy-two hours after transfection, the cells were harvested to detect Sirt2 knockdown by Western blotting.

### Western Blot Analysis

Brain tissues (*n* = 6) or cultured cells were lyzed with lysis buffer (50 mM Tris–HCl, pH 7.6, 150 mM NaCl, 1% Nonidet P-40, 0.5% sodium deoxycholate, 0.1% SDS) containing a protease inhibitor cocktail and the phosphatase inhibitor cocktail solution I (FUJIFILM Wako Pure Chemical Corporation). The resulting lysates were incubated on ice for 30 min and centrifuged at 12,500 rpm for 15 min at 4 °C. The protein concentration was determined using a BCA protein assay kit (Thermo Fisher Scientific). Equal amounts of proteins were separated by SDS-PAGE and transferred to polyvinylidene difluoride membranes (Millipore, Billerica, MA, USA). These membranes were blocked with 5% skim milk or 3% Bovine Serum Albumin (BSA, FUJIFILM Wako Pure Chemical Corporation) in TBS-T for 1 h at RT, and incubated overnight at 4 °C with the following primary antibodies: anti-APP (22C11, 1:1000, Millipore), anti-A disintegrin and metalloproteinase (ADAM)10 (1:1000, Millipore), anti-β-site amyloid precursor protein cleaving enzyme (BACE)1 (1:1000, R&D, Minneapolis, MN, USA), anti-presenilin (PS)1 (1:1000, Millipore), anti-apolipoprotein E (ApoE) (1:1000, Millipore), anti-IDE (1:1000, Covance, Emeryville, CA, USA), anti-neprilysin (NEP) (1:1000, R&D), anti-actin (1:7000, Proteintech, Tokyo, Japan), anti-phosphorylated (p)-tau (T231) (AT180, 1:1000, Invitrogen), anti-p-tau (S202/T205) (PHF, 1:1000, Thermo Fisher Scientific), anti-tau (tau5, 1:1000, BioLegend, San Diego, CA, USA), anti-p-Akt (S473) (1:1000, Cell Signaling Technology), anti-Akt (1:1000, Cell Signaling Technology), anti-p-GSK3α/β (S21/9) (1:1000, Cell Signaling Technology), anti-GSK3α/β (1:1000, Cell Signaling Technology), anti-p-ERK (T202/Y204) (1:1000, Cell Signaling Technology), anti-ERK (1:1000, Cell Signaling Technology), anti-p-JNK (T183/Y185) (1:1000, Cell Signaling Technology), anti-JNK (1:1000, Cell Signaling Technology), anti-p-p38 (T180/Y182) (1:1000, Cell Signaling Technology), anti-p38 (1:1000, Cell Signaling Technology), anti-PP1 (1:1000, Santa Cruz Biotechnology, Dallas, TX, USA), anti-PP2A (1:1000, Abcam, Cambridge, UK), anti-GFAP (1:1000, Sigma-Aldrich), anti-Iba1 (1:1000, Wako), anti-Sirt2 (1:1000, Sigma-Aldrich), and anti-Sirt1 (1:1000, Cell Signaling Technology). The membranes were washed with TBS-T and incubated with the appropriate HRP-conjugated antibodies. Chemiluminescence signals were visualized using Immunostar Zeta or Immunostar LD (FUJIFILM Wako Pure Chemical Corporation) and analyzed using Amersham Imager 680 (GE Healthcare, Marlborough, MA, USA).

### ERK Inhibitor Treatment

To inhibit ERK activity, 1.5 mg ERK inhibitor PD98059 (Cell Signaling Technology) were dissolved in 280 µL anhydrous DMSO to prepare a 20 mM stock solution. The solution was kept at − 20 °C until use. Neuro2a-P301L cells were transfected with Sirt2 or mock control vector for 24 h and treated with 20 µM PD98059 or DMSO for 24 h followed by Western blot analysis.

### Statistical Analysis

Data are presented as mean ± SD for in vivo experiments and presented as mean ± SEM for in vitro experiments of at least three independent experiments. Statistical analysis of the differences among groups was performed with GraphPad Prism (GraphPad Software, San Diego, CA, USA) using one-way ANOVA or Student’s *t* test. Statistical significance was set at *p* < 0.05.

## Results

### STZ-Injected Tg2576 Mice Show High Blood Glucose and Low Serum Insulin Levels

**Fig. 1:**
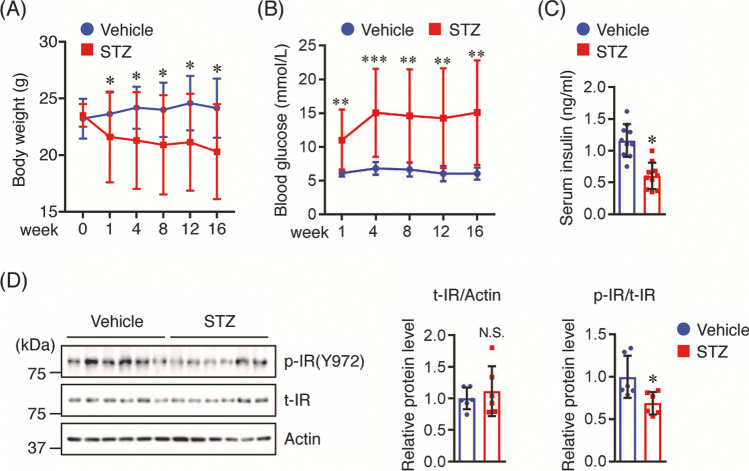
STZ reduces body weight, serum insulin levels, and IR phosphorylation, but enhances blood glucose levels. Three-month-old Tg2576 mice were fasted for 4 h and intraperitoneally (i.p.) injected with STZ (50 mg/kg) or sodium citrate solution only (vehicle control) once a day for 5 consecutive days. **A** Body weight and **B** blood glucose levels were monitored at 1, 4, 8, 12, and 16 weeks after STZ administration (*n* = 11 per group). **C** Sixteen weeks after STZ administration, mice were sacrificed and serum insulin levels were measured using ELISA (*n* = 10 per group). **D** Protein levels of total (t-) and phosphorylated (p-) insulin receptor (IR) in the cortex of mice (*n* = 6 per group) were determined by Western blotting and quantified by densitometry. Quantification of the t-IR and p-IR levels normalized to actin and t-IR levels, respectively, expressed as values relative to the vehicle control. All values are presented as the mean ± SD, *p < 0.05, **p < 0.01, ***p < 0.001, as determined by Student’s *t* test

To clarify the underlying mechanism linking insulin deficiency with AD-like pathologies, we injected 3-month-old Tg2576 mice i.p. with STZ (50 mg/kg) to induce an insulin-deficient AD-like mouse model. Body weight and blood glucose levels were monitored at 1, 4, 8, 12, and 16 weeks after STZ administration. The body weight gradually decreased in STZ-treated mice but slightly increased in age-matched control mice (Fig. 1A). Moreover, the body weight of STZ-treated mice was lower than that of vehicle controls from weeks 4 to 16 (Fig. 1A). Blood glucose levels in STZ-treated mice were higher than those in vehicle controls at all time points examined (Fig. 1B). At 16 weeks after STZ administration, STZ-treated mice showed lower serum insulin levels compared to vehicle controls (Fig. 1C).

### STZ Reduces Insulin Receptor Phosphorylation in the Brains of Tg2576 Mice

Next, we investigated whether STZ administration affected the insulin signaling pathway in the brains of Tg2576 mice. Since insulin binds to the insulin receptor (IR) to exert its biological effects, we measured the levels of both total and phosphorylated (p)-IR. As shown in Fig. 1D, there were no differences in total IR levels between the two groups; in contrast, the p-IR level was lower in the brains of STZ-treated mice than that in vehicle controls. These findings indicate that we succeeded in establishing an insulin-deficient AD-like mouse model in Tg2576 mice by STZ administration, and we used this model to clarify the underlying mechanism linking insulin deficiency with AD-like pathologies.

### STZ Increases Aβ Levels in the Brains of Tg2576 Mice

**Fig. 2:**
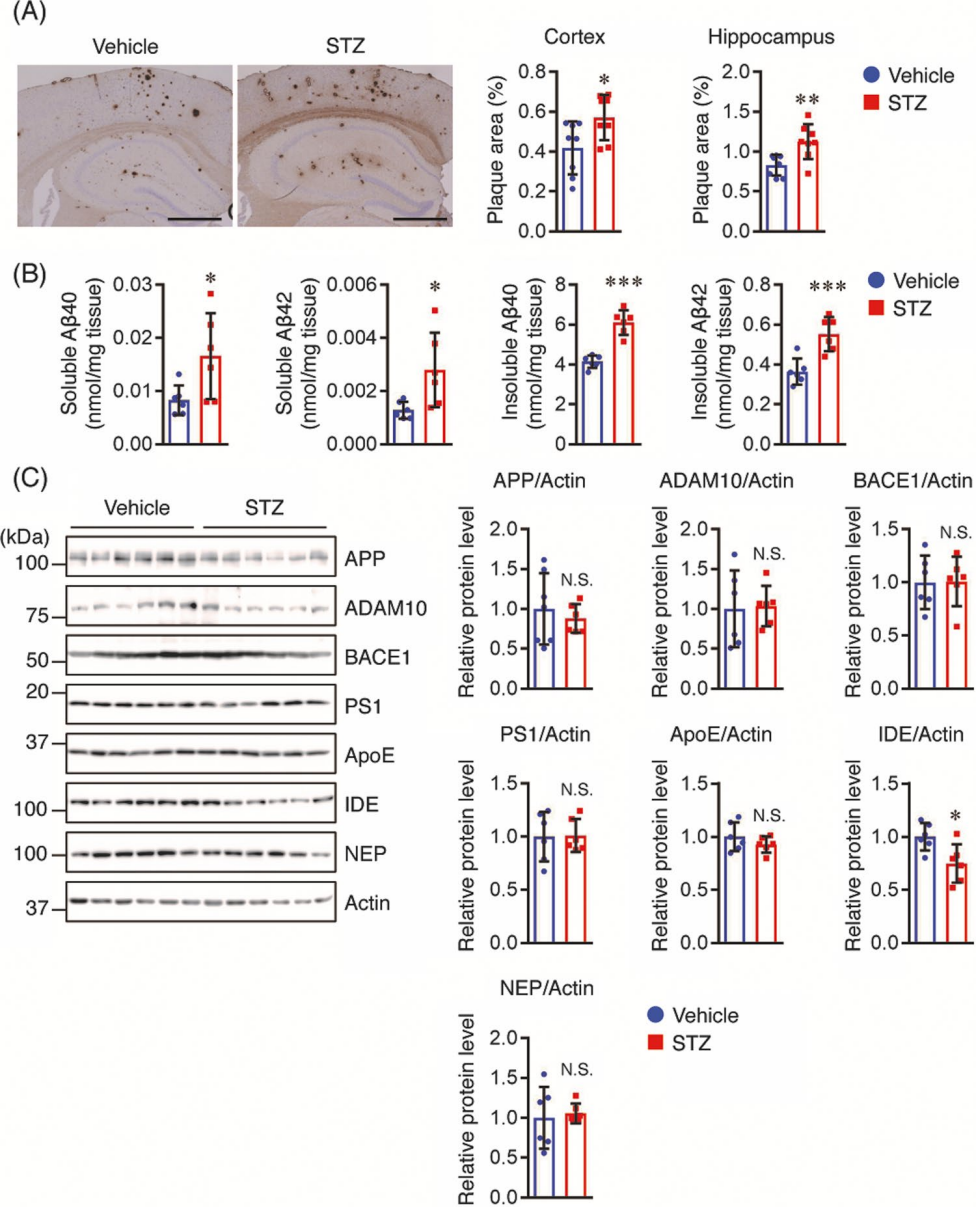
STZ increases Aβ plaque burden and Aβ levels in the hippocampus and cortex of mice. **A** Brain sections (*n* = 8 per group) were stained with anti-Aβ (82E1) antibody recognizing both Aβ40 and Aβ42 to assess Aβ deposition. Representative images are shown on the left panel. Aβ deposits in the cortex and hippocampus were quantified as the percentage of the immunostained area with respect to the total area examined (right panel). Scale bar: 200 μm. **B** Soluble and insoluble Aβ40 and Aβ42 levels in the cortex of mice (*n* = 6 per group) were measured using sandwich ELISA and the levels were normalized to brain tissue weight. **C** The protein levels in the cortex of mice (*n* = 6 per group) were determined by Western blotting, quantified by densitometry, normalized to Actin, and expressed as values relative to the vehicle control. All values are presented as the mean ± SD, *p < 0.05, **p < 0.01, ***p < 0.001, *N*.*S*. no significant difference, as determined by Student’s *t* test

To investigate whether insulin deficiency can affect Aβ levels in the brains of Tg2576 mice, brain sections from vehicle- and STZ-treated Tg2576 mice were stained with anti-Aβ antibody to detect Aβ deposition. We found that the percentages of Aβ-immunopositive areas in the cortex and hippocampus of STZ-treated Tg2576 mice were significantly higher than those in vehicle controls (Fig. 2A). To further validate Aβ deposition, we measured the levels of soluble and insoluble Aβ40 and Aβ42 in the brains of Tg2576 mice using Aβ ELISA. Both soluble and insoluble Aβ40 and Aβ42 levels were markedly higher in STZ-treated mice than those in the vehicle controls (Fig. 2B). To clarify the underlying mechanism by which STZ administration increased Aβ levels in the brains of STZ-treated Tg2576 mice, we assessed the levels of APP and Aβ generation-related proteins, such as ADAM10 (α-secretase), BACE1 (β-secretase), and PS1 (γ-secretase component). However, there were no significant differences in the levels of these proteins between the two groups (Fig. 2C). We also assessed the protein levels of ApoE, an Aβ clearance-related protein, but did not find any changes between the two groups. Next, we measured the protein levels of insulin-degrading enzyme (IDE) and neprilysin (NEP), which are involved in Aβ degradation. Our results showed that the protein levels of IDE in STZ-treated mice were significantly lower than those in vehicle controls, while NEP protein levels were similar between the two groups (Fig. 2C). These findings suggest that the decreased IDE levels in the brains of STZ-treated mice may be attributable to an increase in Aβ levels.

### STZ Induces Tau Hyperphosphorylation in the Brains of Tg2576 Mice

**Fig. 3:**
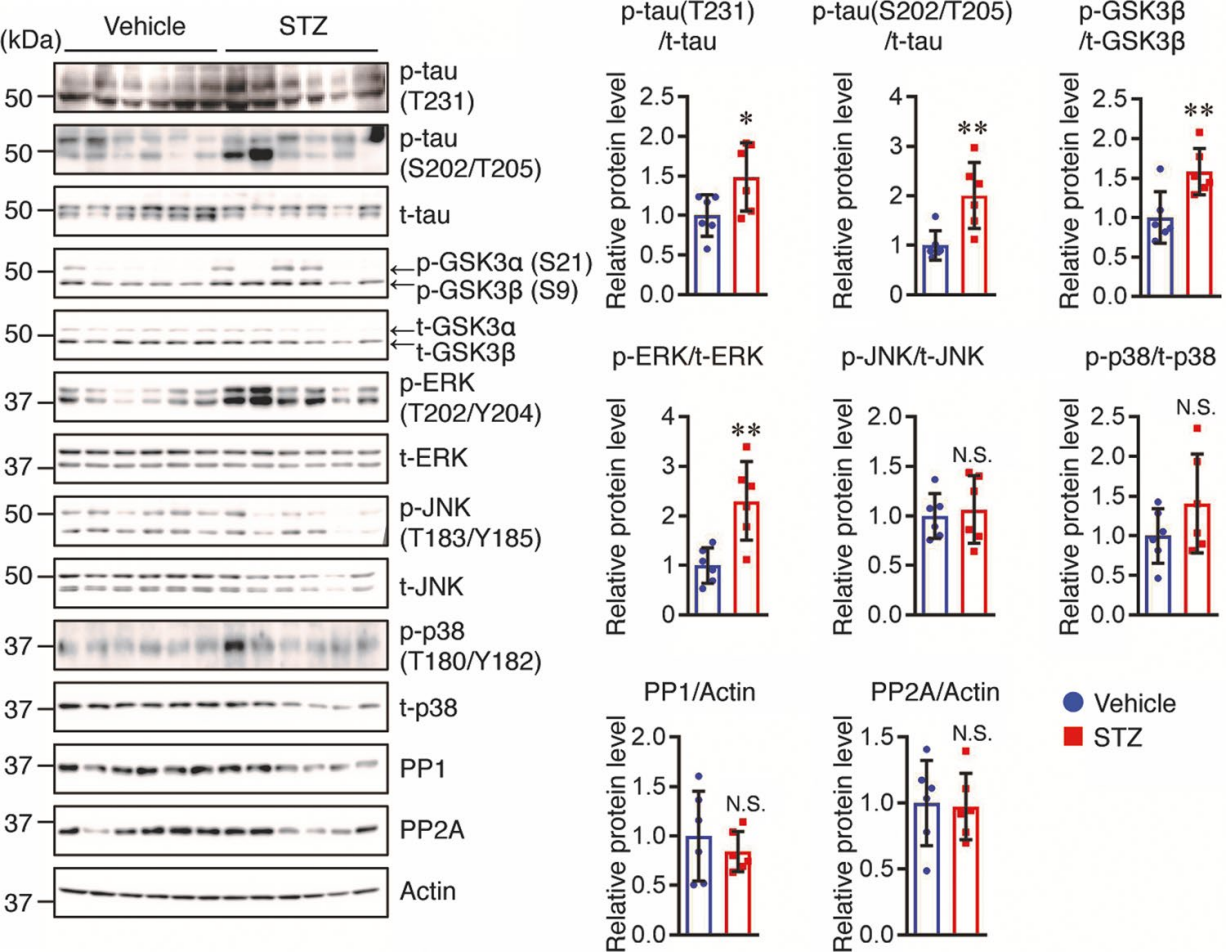
STZ increases the phosphorylation levels of tau and ERK in the cortex of mice. Protein levels in the cortex of mice (*n* = 6 per group) were determined by Western blotting and quantified by densitometry. Quantification of PP1 and PP2A protein levels normalized to Actin. Quantification of phosphorylated (p-) protein levels normalized to total (t-) protein levels, expressed as values relative to the vehicle control. All values are presented as the mean ± SD, *p < 0.05, **p < 0.01, *N*.*S*. no significant difference, as determined by Student’s *t* test

Preclinical studies have demonstrated a link between insulin deficiency and tau pathology, and tau is hyperphosphorylated in the brains of STZ-induced diabetic rats and mice [4, 16]. Therefore, we investigated the phosphorylation levels of tau in our mouse model. We observed an increase in tau phosphorylation at T231 and S202/T205 in the brains of STZ-treated Tg2576 mice compared with that in vehicle controls (Fig. 3). Tau phosphorylation is regulated by a balance between tau kinase and phosphatase levels [20]. Therefore, we assessed the total and phosphorylated levels of tau phosphorylation-associated kinases, such as GSK3β, JNK, ERK, and p38. We also assessed the protein levels of two major tau phosphatases, PP1 and PP2A. We did not find any significant differences in the levels of phosphorylated JNK and p38, or in PP1 and PP2A between the two groups. However, the GSK3β and ERK phosphorylation levels in the brains of STZ-treated mice were higher than those in vehicle controls (Fig. 3). These findings suggest that insulin deficiency induced by STZ enhanced tau hyperphosphorylation through, at least partially, an increase in ERK phosphorylation.

### Insulin Deficiency Induces Neuroinflammation in the Brains of Tg2576 Mice

**Fig. 4:**
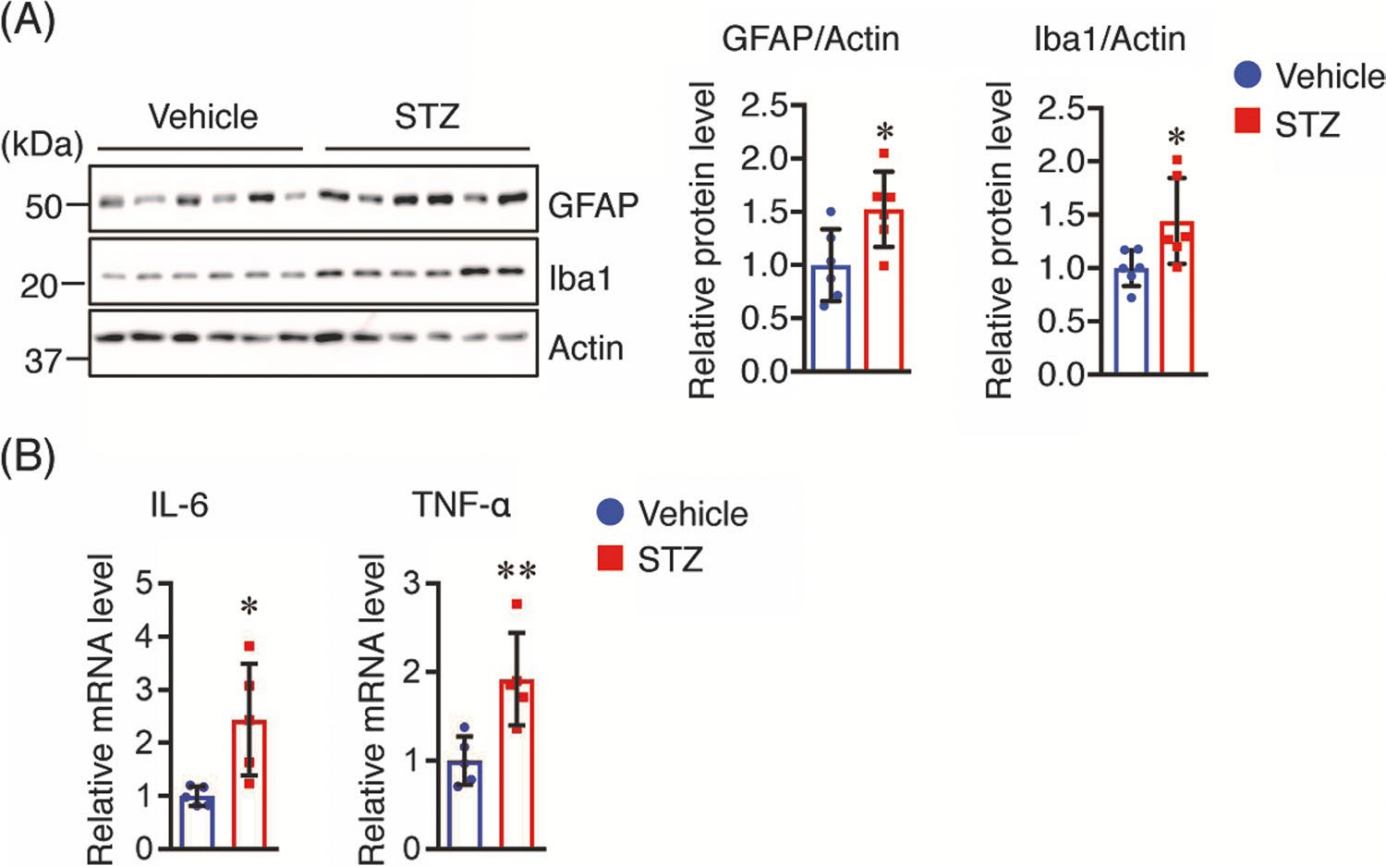
STZ induces neuroinflammation in the brains of mice. **A** GFAP and Iba1 protein levels in the cortex of mice (*n* = 6 per group) was determined by Western blotting, quantified by densitometry, normalized to Actin, and expressed as a value relative to the vehicle control. **B** IL-6 and TNF-α mRNA levels in the cortex of mice (*n* = 5 per group) were determined by real-time PCR analysis. The mRNA levels of IL-6 and TNF-α were normalized to the corresponding expression of GAPDH and expressed as values relative to the vehicle control. All values are presented as the mean ± SD, *p < 0.05, **p < 0.01, as determined by Student’s *t* test

Neuroinflammation is involved in AD pathologies and progression, and insulin deficiency induced by STZ promotes neuroinflammation and glial activation [14], which increases the levels of pro-inflammatory cytokines, including IL-6 and TNF-α [34]. Therefore, we compared the levels of GFAP (a marker of activated astrocytes) and Iba1 (a marker of acivated microglia) between the brains of vehicle- and STZ-treated mice. In STZ-treated mice, both GFAP and Iba1 protein levels were significantly higher than those in vehicle controls (Fig. 4A). In addition, mRNA expression levels of IL-6 and TNF-α in the brains of STZ-treated mice were significantly increased compared to those in vehicle controls (Fig. 4B). These findings suggest that insulin deficiency exacerbates neuroinflammation in the brains of Tg2576 mice.

### Increase in Sirt2 Protein Levels Is Observed in the Brains of STZ-Treated Tg2576 Mice

**Fig. 5:**
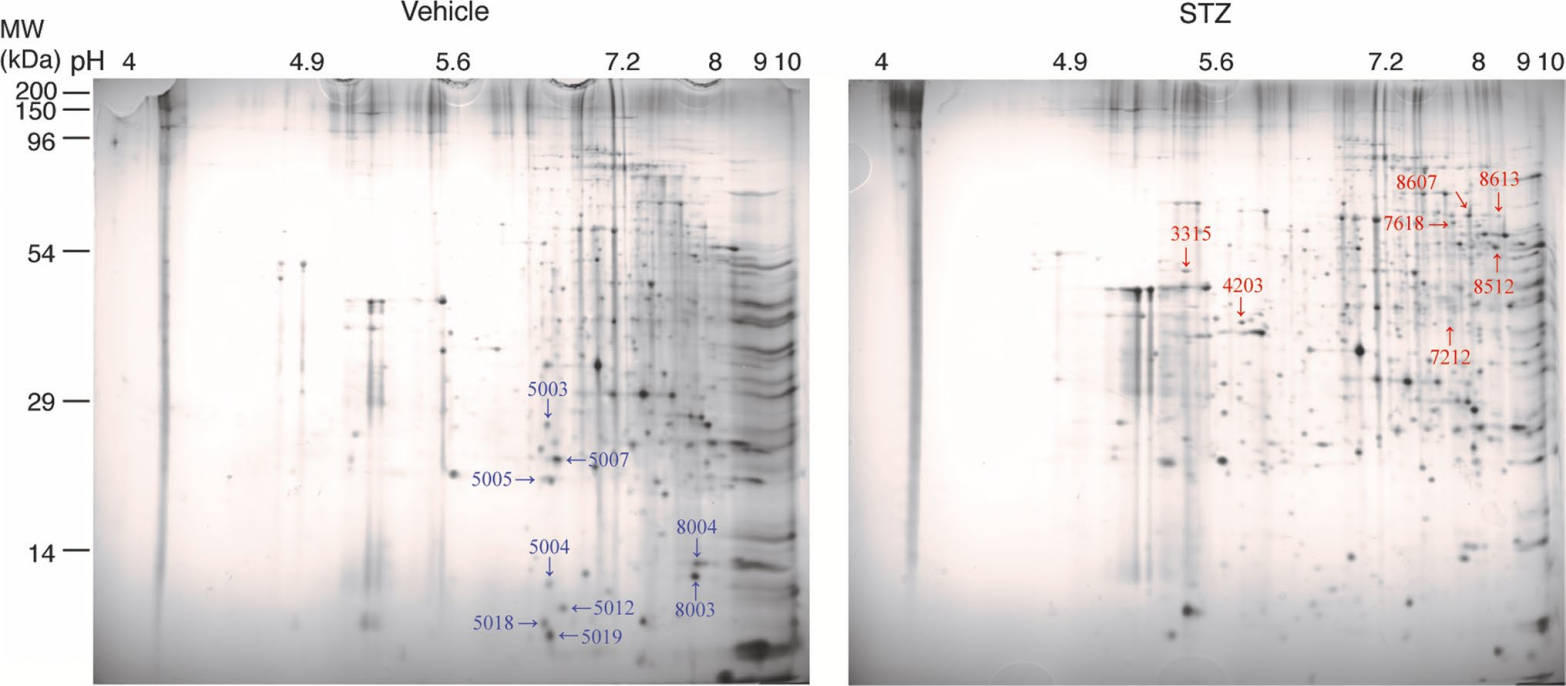
A representative 2-DE gel image of cortical proteins from vehicle- and STZ-treated mice. Cortical proteins obtained from vehicle- and STZ-treated mice were loaded on a pH 4–10, non-linear gel strip for isoelectric focusing and separated using SDS-PAGE. Spots were visualized by Coomassie G250 staining. The blue arrows and numbers indicate downregulated proteins, while the red arrows and numbers indicate upregulated proteins in STZ-treated mice compared with vehicle controls. The spots were selected and identified by LC–MS/MS analysis

**Fig. 6:**
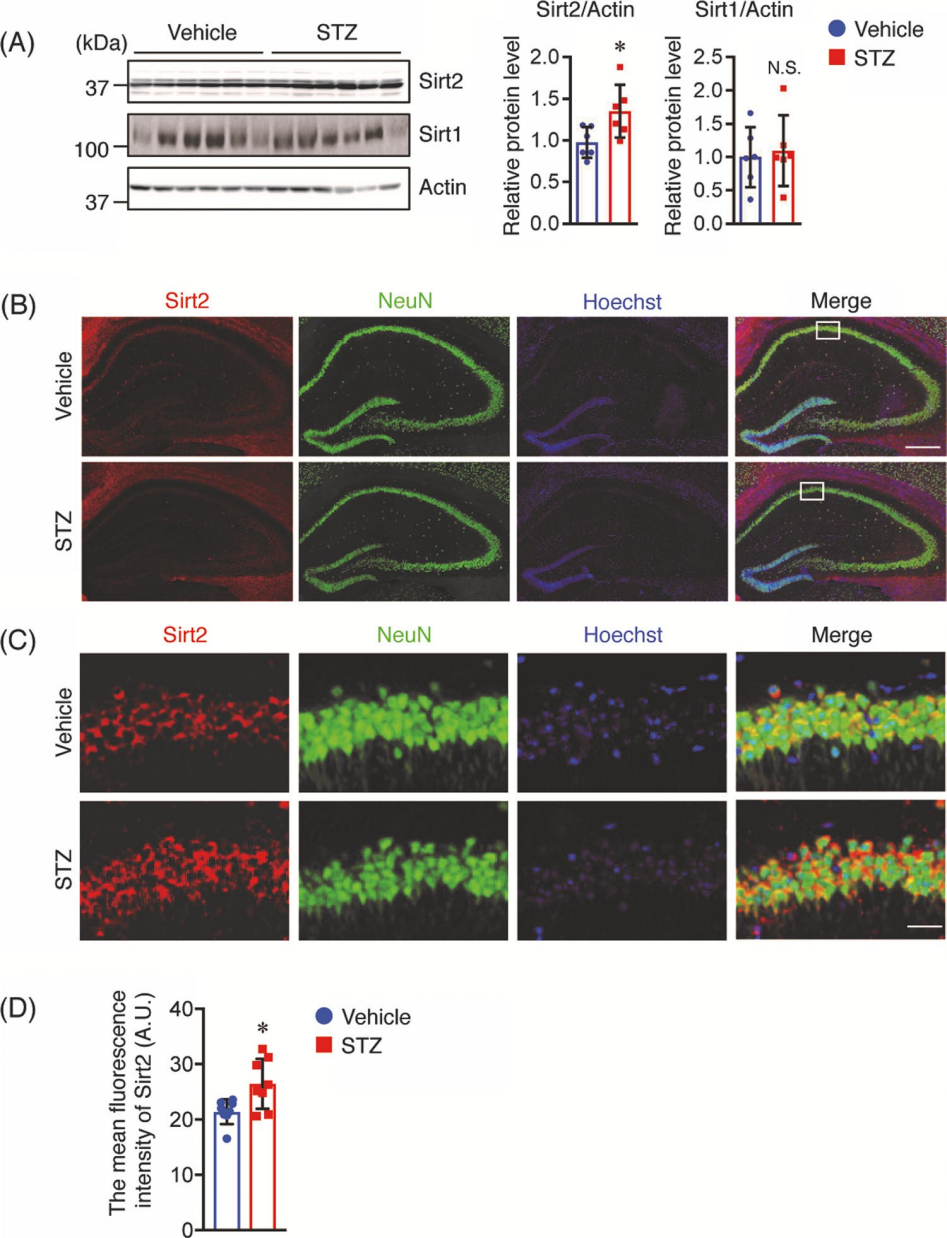
STZ increases Sirt2 levels in the cortex of mice, and Sirt2 is expressed in neurons. **A** Sirt2 and Sirt1 protein levels in the cortex of mice (*n* = 6 per group) were determined by Western blotting, quantified by densitometry, normalized to Actin, and expressed as values relative to the vehicle control. **B** Brain sections were stained with anti-Sirt2 (red) and anti-NeuN (green) antibodies, and cell nuclei were stained with Hoechst. A representative image of the hippocampus is shown. Scale bar: 300 μm. **C** The enlarged view of the white boxes in (**B**). Scale bar: 30 μm. **D** Mean fluorescence intensity of Sirt2-positive cells in the hippocampus (*n* = 8 per group). All values are presented as the mean ± SD, *p < 0.05, *N*.*S*. no significant difference, as determined by Student’s *t* test

To identify the proteins that may affect AD-like pathologies observed in the brains of STZ-treated Tg2576 mice, we performed proteomic analyses of cortical tissues from vehicle- and STZ-treated mice using 2-DE. A total of 16 spots were found to be differentially expressed between vehicle- and STZ-treated mice, seven of which were up- and nine of which were downregulated in STZ-treated mice (Fig. 5). Using LC–MS/MS analysis, we were able to identify the according proteins (Table 1). Among them were mitochondrial proteins, such as cytochrome c oxidase subunit 5B (COX5B), glutamate dehydrogenase 1 (GLUD1), ubiquinol–cytochrome c reductase complex core protein 1 (UQCRC1), and peroxiredoxin 3 (PRDX3), which were altered after STZ administration, indicating that mitochondrial dysfunction was associated with insulin deficiency. Interestingly, we found that Sirt2, an NAD^+^-dependent histone deacetylase, was markedly increased in STZ-treated mice. This finding was corroborated by Western blot analysis (Fig. 6A). Although Sirt2 is known to be involved in neurodegeneration, its role in AD-like pathologies under conditions of insulin deficiency remains unknown. Thus, we focused on the Sirt2 on AD-like pathology under conditions of insulin deficiency. It has been reported that Sirt2 may lead to compensatory activation of Sirt1; however, Sirt1 protein levels were not changed in STZ-treated mice compared with those in vehicle controls (Fig. 6A). Next, to determine the distribution of Sirt2 in the brain, brain sections were stained against Sirt2 and NeuN (neuronal marker) or GFAP (astrocyte marker). The results showed that Sirt2 was expressed in the cytoplasm of NeuN-positive cells both in the hippocampus (Figs. 6 B and C) and cortex (Fig. S2A), but not in GFAP-positive cells in the hippocampus (Fig. S1A and S1B) or the cortex (Fig. S2B). These findings are consistent with previous findings showing that Sirt2 was localized in the cytoplasm of neurons but not in astrocytes [35]. In addition, the mean fluorescence intensities of Sirt2 in the hippocampus (Fig. 6D) and cortex (Fig. S2C) were higher in the STZ-treated mice than in the vehicle control. Taken together, we conclude that the change in neuronal Sirt2 expression may be a pivotal part of the mechanism linking AD-like pathologies with insulin deficiency.

**Table 1:** Differentially expressed cortical protein spots identified by 2-DE and LC–MS/MS between vehicle- and STZ-treated mice

Spot No	Gene name	Protein name	MW (kDa)	pI	Accession No	STZ versus vehicle Ratio
5003		Protease, serine, 1	26.12	6.5	16,716,569	0.27
5004		Unnamed protein product	11.56	6.57	12,838,544	0.08
5005	CDC42 isoform2	Cell division cycle 42 isoform 2	19.42	6.57	16,357,472	0.17
5007	PRDX3	Peroxiredoxin 3	21.74	6.64	6,680,690	0.38
5012	FABP5	Fatty acid binding protein 5	10.58	6.74	6,754,450	0.11
5018	PFN2	Profilin 2	9.66	6.47	9,506,971	0
5019	COX5B	Cytochrome c oxidase subunit 5B	9.04	6.57	117,104	0
8003	NDK1	Nucleoside-diphosphate kinase 1	12.31	8.44	37,700,232	0.64
8004	PPIA	Peptidylprolyl isomerase A	13.08	8.46	6,679,439	0.71
3315	UQCR1	Ubiquinol-cytochrome-c reductase complex core protein 1	49.83	5.35	14,548,301	3.14
4203	DDAH1	Dimethylarginine dimethylaminohydrolase 1	39.32	5.61	14,548,301	1.84
7212	Sirt2	NAD-dependent deacetylase sirtuin-2	40.09	8.23	38,258,618	2.8
7618	CCT6B	T-complex protein 1 subunit zeta-2	66.76	8.3	22,654,292	2.87
8512	GLUD1	Glutamate dehydrogenase 1	56.98	8.88	6,680,027	8.74
8607	STXBP1	Syntaxin binding protein 1	69.98	8.53	4,507,297	3.74
8613	HNRNPL	Heterogeneous nuclear ribonucleoprotein L	69.57	8.93	46,577,278	6.79

### Insulin Depletion and IL-6 Treatment Increase Sirt2 Protein Levels

**Fig. 7:**
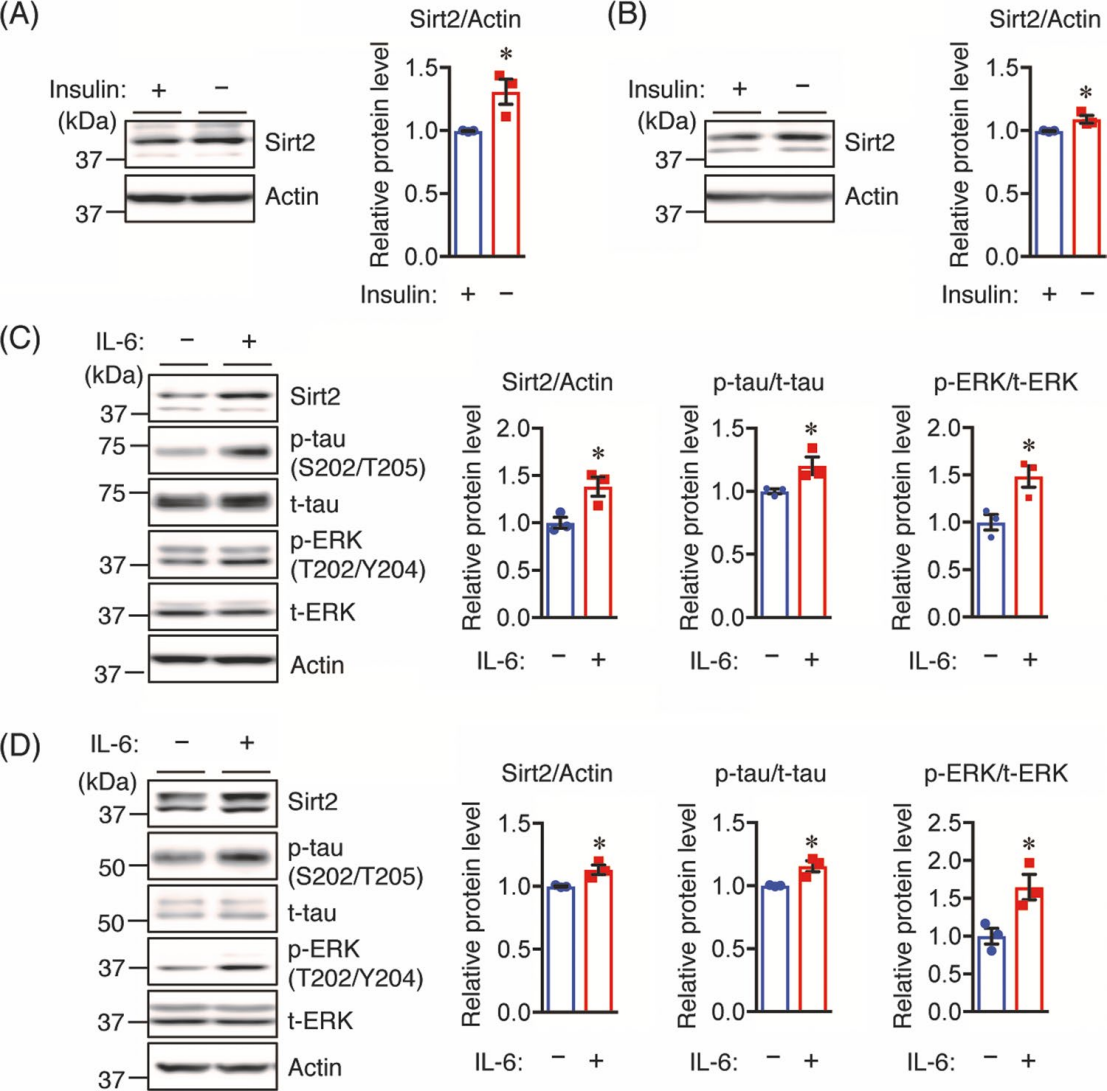
Insulin depletion or IL-6 increases the Sirt2 protein level. **A** Neuro2a-P301L and **B** Neuro2a cells were treated with or without insulin (10 nM) in FBS-free medium for 24 h before lysis. **C** Neuro2a-P301L and **D** Neuro2a cells were treated with 50 ng/mL IL-6 or vehicle control in FBS-free medium for 30 min before lysis. Protein levels were determined by Western blotting and quantified by densitometry. Quantification of Sirt2 protein levels normalized to Actin. Quantification of phosphorylated (p-) protein levels normalized to total (t-) protein levels and expressed as values relative to the control. All values are presented as the mean ± SEM, *p < 0.05, as determined by Student’s *t* test; *n* = 3 per group

To understand the underlying mechanism of increased Sirt2 levels observed in the brains of STZ-treated mice, we investigated the effect of insulin depletion on Sirt2 protein levels in Neuro2a and Neuro2a-P301L cells stably expressing human tau with the P301L mutation using Western blotting. We found that insulin depletion increased Sirt2 levels in both Neuro2a-P301L (Fig. 7A) and Neuro2a (Fig. 7B) cells. Based on our findings that IL-6 and TNF-α were increased in the brains of STZ-treated mice (Fig. 4B), we hypothesized that these inflammatory cytokines may have induced Sirt2 expression. To this end, we treated Neuro2a-P301L and Neuro2a cells with 50 ng/mL IL-6 for 30 min and measured protein levels of Sirt2 levels using Western blotting. Compared with the controls, IL-6 treatment increased Sirt2 levels in both Neuro2a-P301L (Fig. 7C) and Neuro2a cells (Fig. 7D). Furthermore, we found that IL-6 treatment increased phosphorylation levels of tau and ERK in both Neuro2a-P301L (Fig. 7C) and Neuro2a cells (Fig. 7D), which is consistent with previous findings showing that IL-6 is involved in the induction of tau and ERK phosphorylation in neuronal cells [36]. Taken together, these findings suggest that the increased Sirt2 levels observed in STZ-treated mice may be caused by both insulin deficiency and increased IL-6 levels.

### Overexpression of Sirt2 Increases Tau Phosphorylation Levels and ERK Activation

**Fig. 8:**
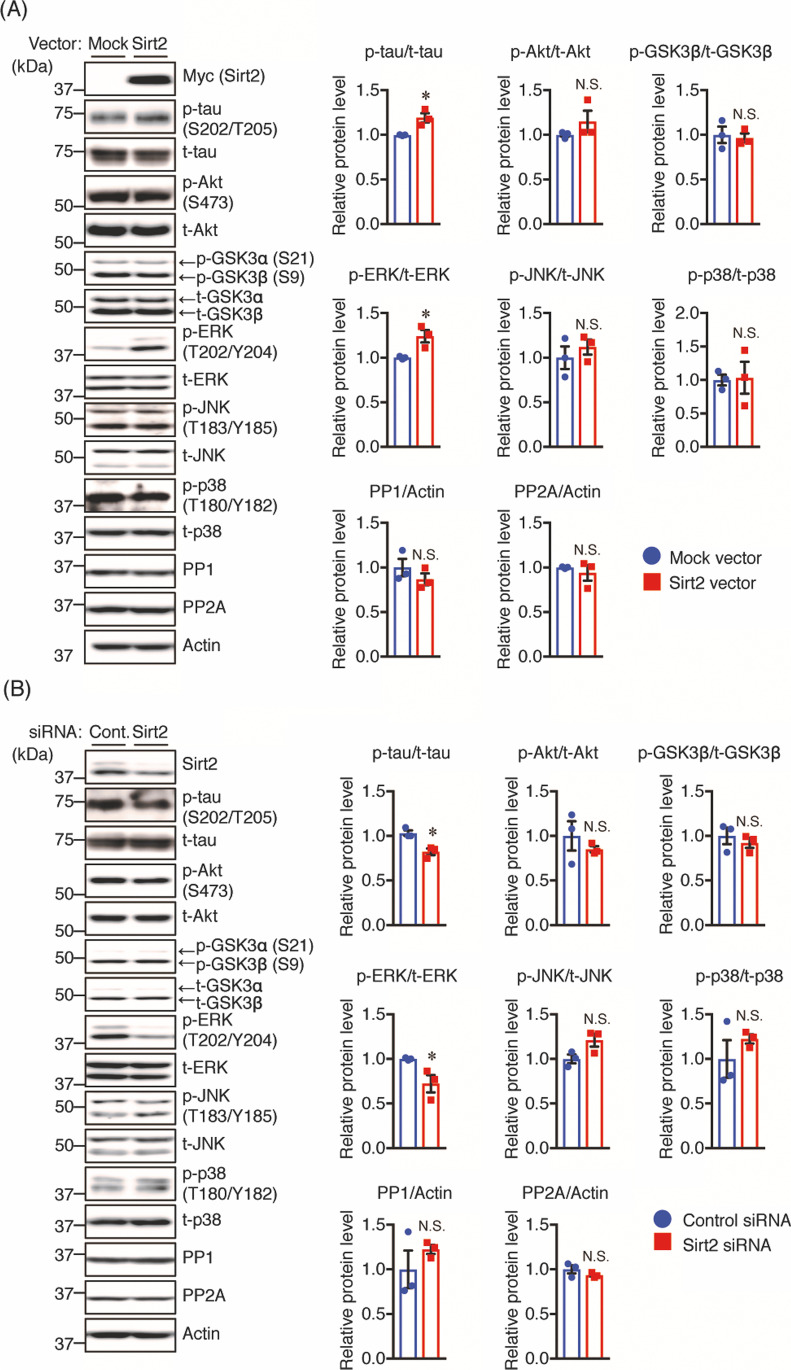
Sirt2 regulates the phosphorylation levels of tau and ERK in Neuro2a-P301L cells. **A** Neuro2a-P301L cells were transfected with mock control or Sirt2 vector for 48 h before lysis. **B** Neuro2a-P301L cells were transfected with the control or Sirt2 siRNA for 72 h before lysis. Protein levels were determined by Western blotting and quantified by densitometry. Quantification of PP1 and PP2A protein levels normalized to Actin. Quantification of phosphorylated (p-) protein levels normalized to total (t-) protein levels and expressed as values relative to the control. All values are presented as the mean ± SEM, *p < 0.05, *N*.*S*. no significant difference, as determined by Student’s *t* test; *n* = 3 per group

Given our findings of increased tau phosphorylation and Sirt2 levels upon STZ treatment, we investigated the effects of Sirt2 on tau phosphorylation. We overexpressed Sirt2 in Neuro2a-P301L or Neuro2a cells and assessed the levels of total and p-tau using Western blotting. We found that overexpression of Sirt2 enhanced tau phosphorylation at S202/T205 in both Neuro2a-P301L (Fig. 8A) and Neuro2a (Fig. S3A) cells. To study the mechanisms underlying the enhancement of tau phosphorylation induced by Sirt2, we measured the levels of tau phosphorylation-associated proteins. We found that the overexpression of Sirt2 did not alter the levels of phosphorylated Akt, GSK3β, JNK, and p38, and neither were PP1 or PP2A changed in Neuro2a-P301L cells compared with controls (Fig. 8A). In contrast, we found that Sirt2 overexpression enhanced ERK phosphorylation in both Neuro2a-P301L (Fig. 8A) and Neuro2a (Fig. S3A) cells compared with controls.

### Knockdown of Sirt2 Decreases Tau Phosphorylation and ERK Activation

Next, we examined the effect of Sirt2 knockdown on tau phosphorylation. After Neuro2a-P301L cells were transfected with control or Sirt2 siRNA, total and phosphorylated tau levels were determined using Western blotting. As shown in Fig. 8B, Sirt2 knockdown significantly decreased p-tau and p-ERK levels. In addition, there was no change in the levels of phosphorylated Akt, GSK3β, JNK, and p38 between Sirt2 siRNA-transfected and control cells; the levels of PP1 and PP2A were also unchanged. We further tested whether Sirt2 knockdown decreased tau phosphorylation in Neuro2a cells and primary cultured neurons. Consistent with the results of Neuro2a-P301L cells, Sirt2 knockdown decreased the phosphorylation levels of tau and ERK in Neuro2a cells (Fig. S3B) and primary cultured neurons (Fig. S4). The phosphorylation levels of Akt, GSK3β, JNK, and p38 were not changed in Sirt2 siRNA-transfected primary cultured neurons (Fig. S4).

### Sirt2 Regulates Tau Phosphorylation Levels via ERK Activation

**Fig. 9:**
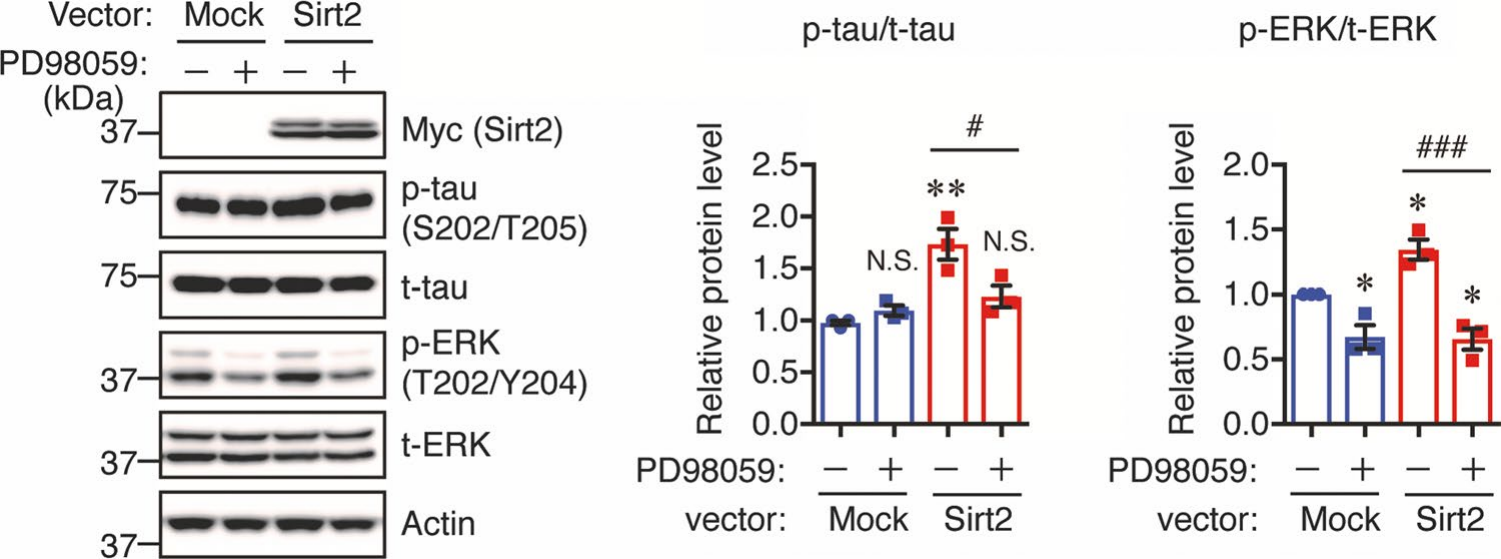
ERK inhibitor suppresses Sirt2-mediated tau phosphorylation in Neuro2a-P301L cells. Neuro2a-P301L cells were transfected with mock control or Sirt2 vector for 24 h, treated with 20 µM ERK inhibitor (PD98059) for 24 h, and then lysed. Protein levels were determined by Western blotting and quantified by densitometry. Quantification of phosphorylated (p-) protein levels normalized to total (t-) protein levels and expressed as values relative to the control. All values are presented as the mean ± SEM, *p < 0.05, **p < 0.01, ^#^*p* < 0.05, ^###^*p* < 0.001, *N*.*S*. no significant difference, as determined by one-way ANOVA; *n* = 3 per group

Based on our Sirt2 overexpression and knockdown experiments, we hypothesized that Sirt2 may regulate tau phosphorylation via ERK activity. To confirm that ERK activity is involved in Sirt2-mediated tau phosphorylation, Neuro2a-P301L cells were transfected with mock control or Sirt2 vector for 24 h and treated with vehicle or 20 µM ERK inhibitor for 24 h. We found that the inhibition of ERK prevented Sirt2-mediated tau phosphorylation (Fig. 9). Taken together, these results suggest that Sirt2 mediates tau phosphorylation at least in part via the activation of ERK.

## Discussion

Insulin deficiency and resistance are strongly associated with memory deficits and cognitive decline, and AD shares molecular and cellular features with T1DM and T2DM, suggesting that AD may be a type 3 diabetes [6]. In this study, we subjected Tg2576 mice to STZ to produce combined insulin-deficient diabetes and AD. We found that STZ markedly increased blood glucose levels, decreased serum insulin levels, and reduced phosphorylated IR levels in the brains of these mice. In addition, STZ increased Aβ levels, tau hyperphosphorylation, and neuroinflammation in the brains of Tg2576 mice. We found for the first time that STZ increased Sirt2 protein levels in the mouse brain. Furthermore, our in vitro experiments revealed that insulin depletion or IL-6 treatment increased Sirt2 levels, and the increased Sirt2 levels induced tau hyperphosphorylation through ERK activation. These findings suggest that Sirt2 is involved in insulin deficiency-induced tau hyperphosphorylation.

Consistent with previous findings showing that insulin deficiency exacerbated the accumulation of Aβ in the brains of STZ-treated APP transgenic mice [17, 18], our study also demonstrated that STZ increased soluble and insoluble Aβ levels as well as Aβ deposition in the mouse brain. The mechanism underlying the STZ-mediated increase in Aβ generation is controversial. While some studies have shown that STZ increased BACE1 levels, leading to Aβ generation in the brains of APP/PS1 mice [18], other studies revealed that STZ increased Aβ generation independently of BACE1 in APP transgenic mice and a monkey model of T1DM induced by injection of STZ [17, 37]. In this study, we did not find any significant changes in the levels of APP and Aβ generation-related proteins, such as ADAM10, BACE1, and PS1, between vehicle- and STZ-treated mice, indicating that the increased Aβ levels may not have been caused by accelerated Aβ generation in our mouse model. Insulin deficiency decreases the expression level of IDE [38], a major Aβ-degrading enzyme, which is negatively correlated with Aβ degradation, thus contributing to an increase in Aβ levels in the brain [39]. Consistent with a previous report demonstrating that STZ administration decreased IDE protein levels, resulting in increased Aβ levels in APP transgenic mice [17], our study also showed that IDE levels in the brains of STZ-treated mice were lower than those in vehicle controls. Thus, the increase in Aβ levels in the brains of STZ-treated mice may have been caused by the impaired ability of Aβ degradation through downregulation of IDE levels.

Tau is hyperphosphorylated at multiple sites in the mouse brain in response to STZ-induced insulin deficiency, including Thr231, Thr205, and Ser202 [4]. Here, we confirmed that STZ increased the levels of hyperphosphorylated tau at Thr231 and Ser202/Thr205. Tau phosphorylation levels are regulated by the balance between tau kinases and phosphatases. GSK3β has been proposed to play a key role in tau phosphorylation, and several studies have shown that the activation of GSK3β is linked to the mechanisms by which STZ causes tau phosphorylation in APP transgenic mouse models [17, 18]. In contrast, some studies have shown that although STZ injection induced an increase in tau phosphorylation, inhibitory phosphorylation at Ser9 in GSK3β was increased in APP transgenic and non-transgenic mice [14, 40]. In this study, we also found that STZ injection increased inhibitory phosphorylation at Ser9 in GSK3β, indicating that GSK3β activity may not be directly involved in tau phosphorylation in our mouse model. We further investigated the potential involvement of tau kinases, such as ERK, JNK, and p38, which are major kinases downstream of the insulin signaling pathway [41, 42] and the overactivation of which could accelerate tau phosphorylation [43].We found that while JNK and p38 activation did not change, ERK activation was enhanced in the brains of STZ-treated Tg2576 mice, which is consistent with the previous finding that ERK activation was enhanced in the brains of STZ-injected mice [44]. Our results therefore suggest that STZ-induced ERK activation may be at least partially responsible for tau hyperphosphorylation in AD-like pathology.

A recent study has shown that mitochondrial function is impaired in STZ-induced diabetic rodents [45, 46], and our study also showed that the expression of some mitochondrial proteins was changed after STZ administration. COX is an enzyme in the respiratory electron transport chain, and mammalian COX is composed of 14 subunits [47]. In patients with late-onset AD, COX5A, COX5B, COX7A2, COX7A2L, and CYC1 are downregulated, resulting in depleted energy provision [48]. Glutamate dehydrogenase, encoded by *GLUD1*, participates in the breakdown and synthesis of glutamate, which is the major excitatory neurotransmitter in the brain [49]. Abnormalities in glutamate maintenance are associated with several neurodegenerative diseases, such as PD and AD [49]. UQCRC1 is a component of mitochondrial complex III, the assembly of which is downregulated in early-onset AD patients [48]. PRDXs are a family of thiol-dependent peroxidases that catalyze the reduction of hydrogen peroxide, peroxynitrite, and alkyl hydroperoxides [50]. In the brains of patients with AD, PRDX3 is downregulated compared to age-matched controls [51]. Here, the altered levels of enzymes involved in energy production, neurotransmitter synthesis, and reactive oxygen species (ROS) reduction indicate an impairment of mitochondrial function in our insulin-deficient AD-like mouse model.

High levels of Sirt2 are observed in neurodegenerative disorders, including AD and PD [22], suggesting that Sirt2 inhibition may be a therapeutic strategy for the treatment of such diseases; this is further substantiated by the fact that Sirt2 inhibitors prevent neuronal loss [52]. However, the expression and involvement of Sirt2 in AD pathology under conditions of insulin deficiency is still unknown. In this study, we found for the first time that Sirt2 protein levels were increased in the brains of STZ-treated Tg2576 mice. Although the regulatory mechanism of Sirt2 expression is still unknown, Aorora et al. reported that insulin resistance increased Sirt2 levels in C2C12 skeletal muscle cells, indicating that the dysfunction of insulin signaling may induce Sirt2 expression [53]. Therefore, we considered that the increased Sirt2 levels in the brains of STZ-treated mice could be due to insulin deficiency, and found that insulin depletion in both Neuro2a-P301L and Neuro2a cells significantly increased Sirt2 protein levels. Moreover, it has been reported that oxidative stress increases Sirt2 levels in 293 T cells and adipocytes [54] and oxidative stress-activated astrocytes and microglia, which leads to the induction of pro-inflammatory cytokines, such as IL-6 and TNF-α [34]. Here, we found that IL-6 treatment increased Sirt2 levels in both Neuro2a-P301L and Neuro2a cells. Taken together, the upregulated Sirt2 levels in the brains of STZ-treated mice may be due to both insulin deficiency and increased IL-6 levels produced by activated astrocytes. Additional studies are required to determine the mechanisms by which insulin deficiency and IL-6 increase Sirt2 expression.

Several lines of evidence have shown that Sirt2 inhibitor (AGK-2 and AK-7) injection in AD-like mouse models decreased Aβ generation by reducing BACE1 levels, thus leading to an amelioration of cognitive impairment [26, 55]. Although the function of Sirt2 in Aβ pathology has been well investigated, the involvement of this enzyme in tau phosphorylation has not been fully examined. Thus, we focused on the effect of Sirt2 on tau phosphorylation in in vitro experiments. ERK is part of one of the downstream pathways of insulin signaling and its activation is responsible for tau hyperphosphorylation both in vitro and in vivo [56, 57]. Here, we found that overexpression of Sirt2 in both Neuro2a-P301L and Neuro2a cells increased tau phosphorylation, whereas knockdown of Sirt2 in these cells and in primary neurons decreased tau phosphorylation. To investigate the mechanisms underlying these effects, we examined the phosphorylation levels of tau kinases, such as GSK3β, JNK, ERK, and p38. Interestingly, we found that overexpression of Sirt2 significantly enhanced the level of ERK phosphorylation, but had no effect on the phosphorylation levels of the other kinases analyzed. Conversely, the reduction of Sirt2 by knockdown decreased ERK phosphorylation. Furthermore, treatment of Sirt2-overexpressed Neuro2a-P301L cells with an ERK inhibitor prevented Sirt2-mediated tau hyperphosphorylation, suggesting that ERK activity is involved in Sirt2-mediated tau phosphorylation. These findings are consistent with a previous report indicating that Sirt2 overexpression in human gastric cell lines increased ERK phosphorylation [58]. Thus, we suggest that an increased Sirt2 level induces tau hyperphosphorylation in the brains of STZ-treated mice via ERK activation. It has been reported that ERK can bind to Sirt2, leading to increased stability and activity of Sirt2 [59], while Sirt2 expression positively associated with ERK phosphorylation in some cancer cell lines [58, 60]. Although these findings indicated that Sirt2 and ERK are mutually regulated, the underlying mechanism by which Sirt2 is directly involved in ERK activation is unclear. Several lines of evidence reported that Sirt2 knockdown may inactivate the RAS/ERK signaling cascade. For example, Sirt2 knockdown deacetylated KRAS, which could further reduce ERK phosphorylation [61]. Further studies are required to identify metabolic downstream targets of Sirt2 that may contribute ERK activation and to further investigate the mechanisms underlying the relationships between Sirt2 and ERK.

## Conclusions

In this study, we found that STZ-induced T1DM-like Tg2576 mice showed exacerbated amyloidosis, tau hyperphosphorylation, and neuroinflammation. Sirt2 expression levels were upregulated in the brains of these mice, and Sirt2 promoted tau phosphorylation via ERK activity in vitro. Our study is the first to characterize the regulation of Sirt2 expression and its impact on tau phosphorylation under insulin deficiency. While further investigation will be required to clarify the complex role of Sirt2 and ERK in AD, Sirt2 may be a suitable target for therapeutic interventions in this disease.

## Supplementary Information

Below is the link to the electronic supplementary material.

Supplementary file1 (PDF 1106 KB)

## Data Availability

All data used in this study are available from the corresponding authors on reasonable request.
