# Supplementary material for: Insulin Deficiency Increases Sirt2 Level in Streptozotocin-Treated Alzheimer’s Disease-Like Mouse Model: Increased Sirt2 Induces Tau Phosphorylation Through ERK Activation
Source: Mol Neurobiol. 2022 Jun 15;59(9):5408–25. doi: 10.1007/s12035-022-02918-z (PMC9395464; doi:10.1007/s12035-022-02918-z)
Supplement: Supplementary file 1 — Supplementary file1 (PDF 1106 KB) [file 12035_2022_2918_MOESM1_ESM.pdf]

Additional files: Supplementary Figure legends

Supplementary Fig. S1. Sirt2 is not expressed in GFAP-positive astrocytes in mouse hippocampus.
(A) Brain sections were stained with anti-Sirt2 (red) and anti-GFAP (green) antibodies, and cell nuclei were stained with Hoechst. A representative image of the hippocampus is shown. Scale bar: 300 μm. (B) The enlarged view of the white boxes in (A). Scale bar: 30 μm.

Supplementary Fig. S2. Sirt2 is expressed in NeuN-positive neurons, but not in GFAP-positive astrocytes in the cortex.
(A) Brain sections were stained with anti-Sirt2 (red) and anti-NeuN (green) antibodies, and cell nuclei were stained with Hoechst. A representative image of the cortex is shown. Scale bar: 150 μm. The enlarged view of the white boxes is shown in the right panel. Scale bar: 15 μm. (B) Brain sections were stained with anti-Sirt2 (red) and anti-GFAP (green) antibodies, and cell nuclei were stained with Hoechst. A representative image of the cortex is shown. Scale bar: 150 μm. The enlarged view of the white boxes is shown in the right panel. Scale bar: 15 μm. (C) Mean fluorescence intensity of Sirt2-positive cells in the cortex ($n = 8$ per group). All values are presented as the mean ± SD, *$p < 0.05$, as determined by Student's *t*-test.

Supplementary Fig. S3. Sirt2 regulates the phosphorylation levels of tau and ERK in Neuro2a cells.

(A) Neuro2a cells were transfected with mock control or Sirt2 vector for 48 h before lysis. (B) Neuro2a cells were transfected with the control or Sirt2 siRNA for 72 h before lysis. Protein levels were determined by western blotting and quantified by densitometry. Quantification of phosphorylated (p-) protein levels normalized to total (t-) protein levels and expressed as values relative to the control. All values are presented as the mean ± SEM, **p* < 0.05, ****p* < 0.001, as determined by Student's *t*-test; n = 3 per group.

Supplementary Fig. S4. Sirt2 knockdown reduces the phosphorylation levels of tau and ERK in primary cultured neurons.

Primary cultured neurons were transfected with control or Sirt2 siRNA for 72 h before lysis. Protein levels were determined by western blotting and quantified by densitometry. Quantification of Sirt2 protein levels normalized to Actin. Quantification of phosphorylated (p-) protein levels normalized to total (t-) protein levels and expressed as values relative to the control siRNA. All values are presented as the mean ± SEM, *p < 0.05, ****p* < 0.001, N.S., no significant difference, as determined by Student's *t*-test; n = 3 per group.

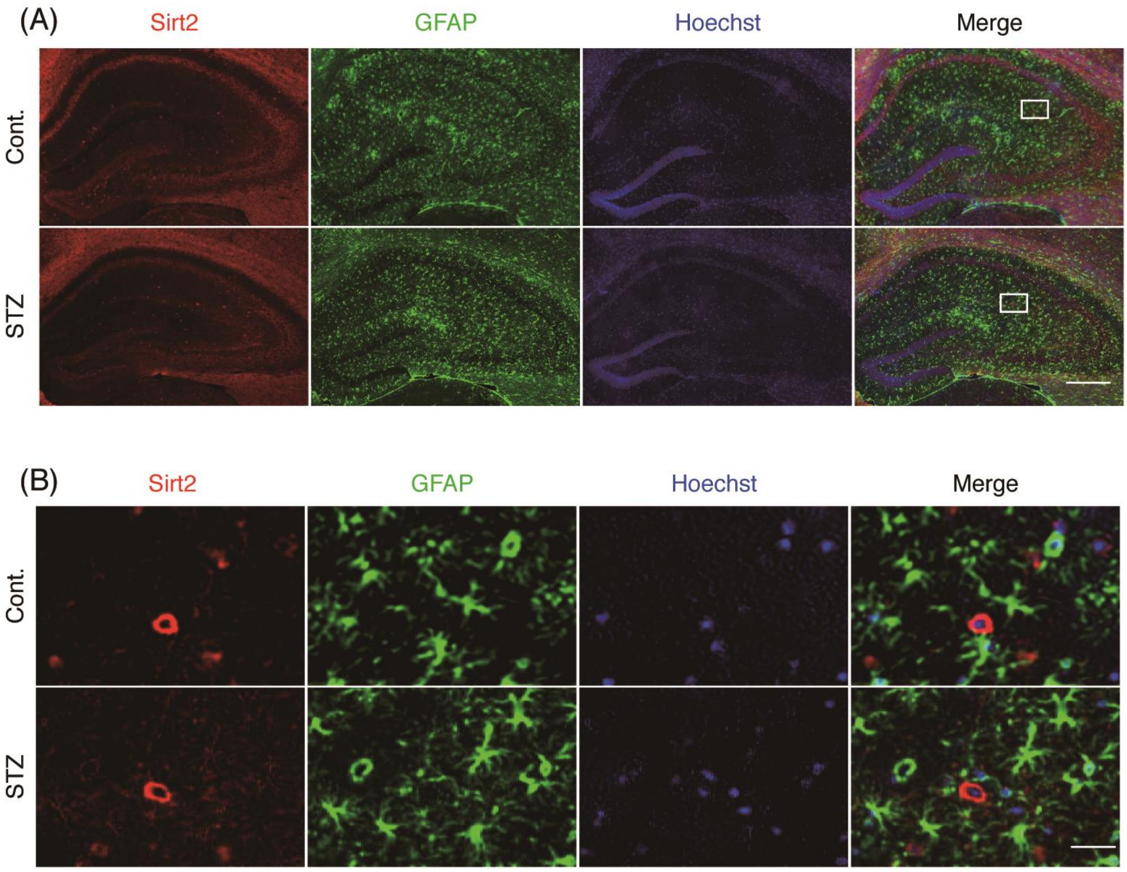

Supplementary Fig. S1.

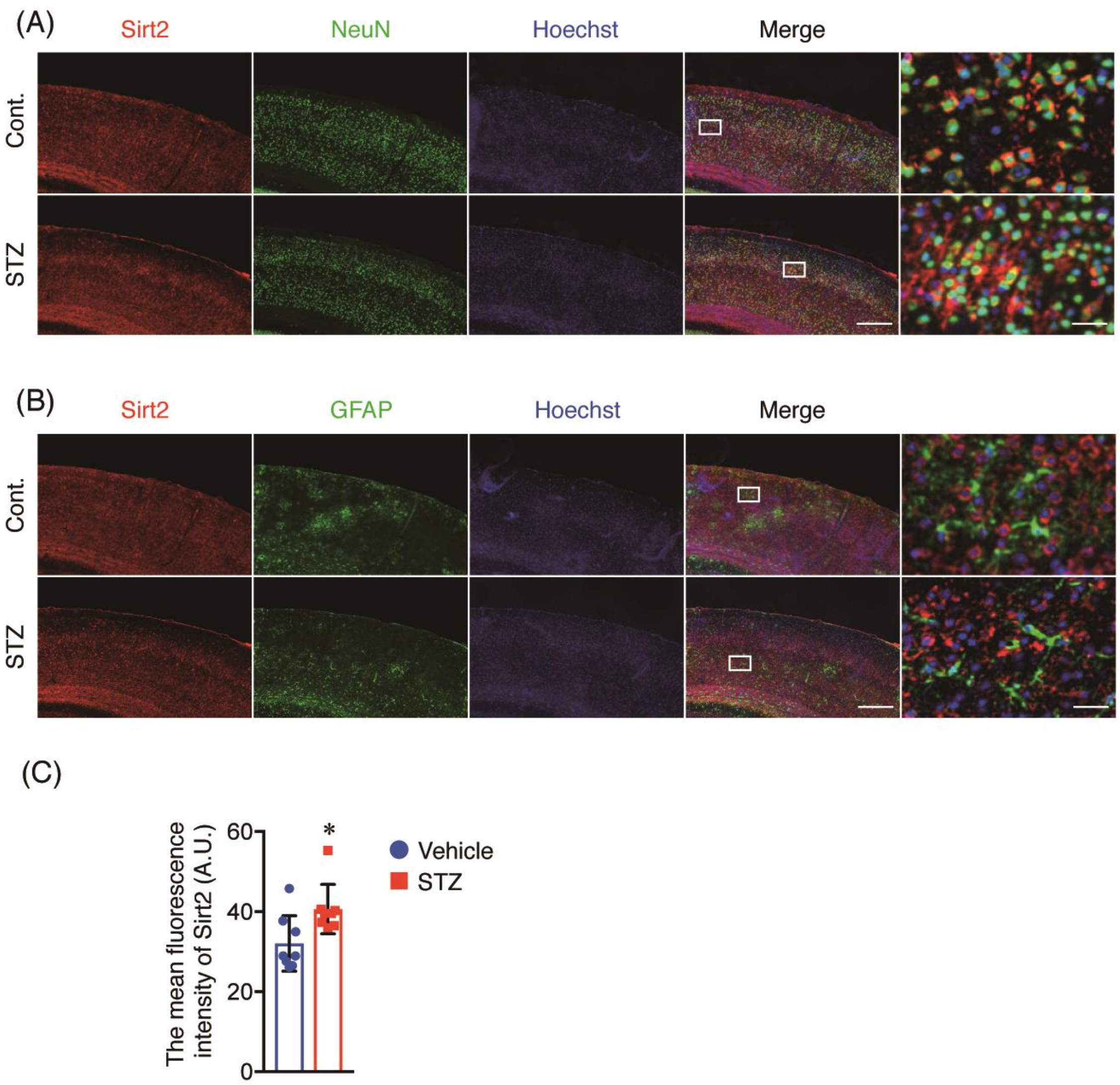

Supplementary Fig. S2.

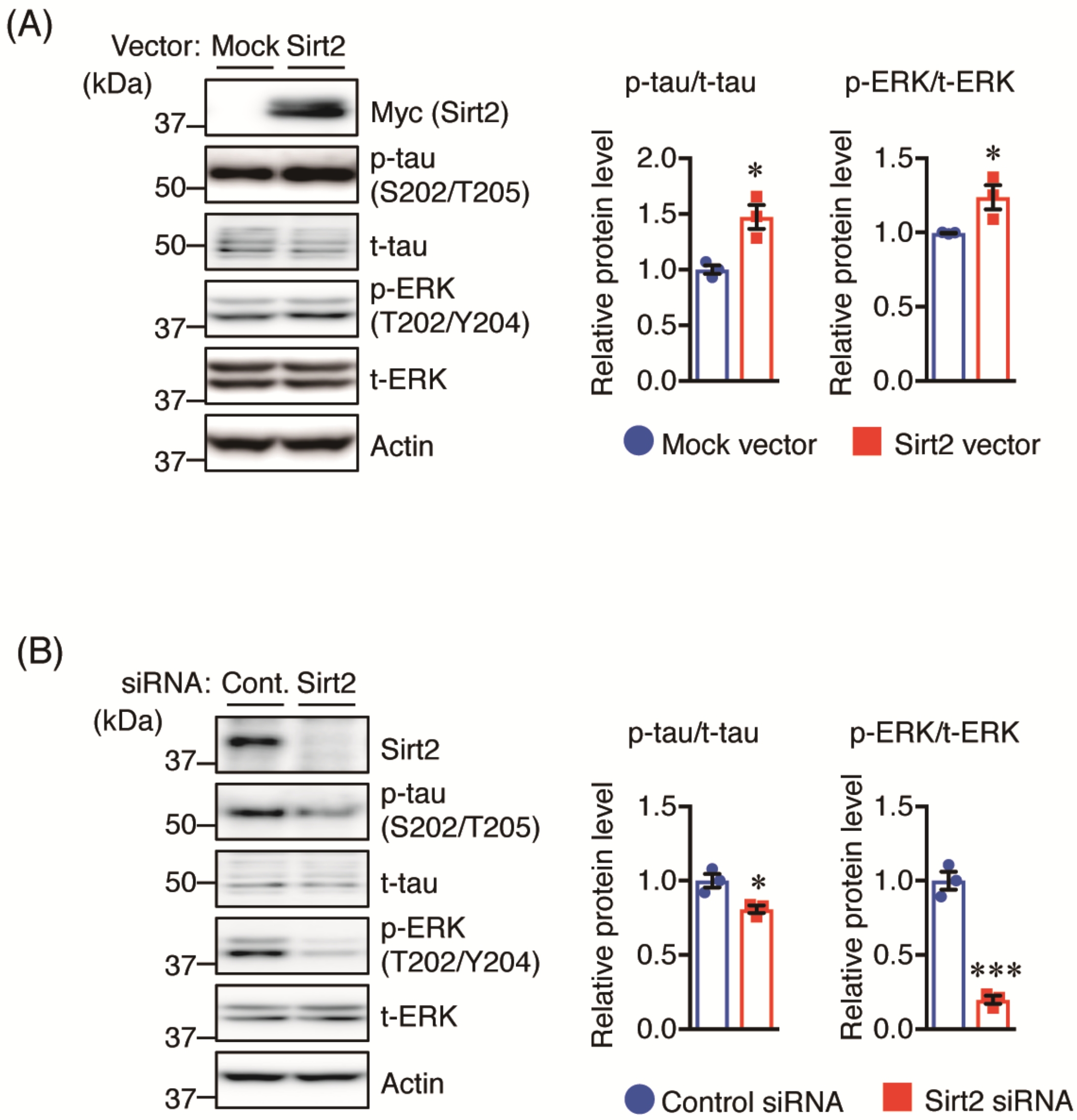

Supplementary Fig. S3.

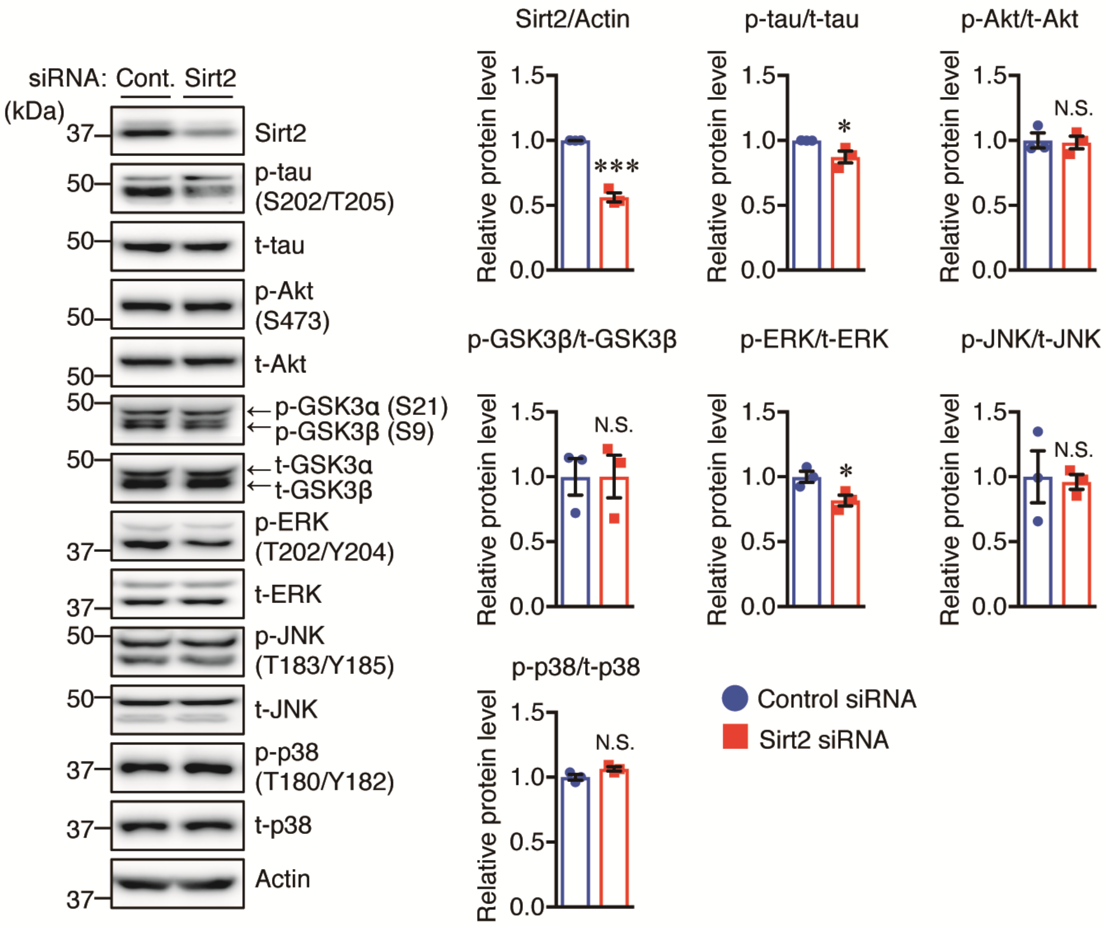

Supplementary Fig. S4.
